# Supplementary material for: Gene expression profiling of patient‐derived pancreatic cancer xenografts predicts sensitivity to the BET bromodomain inhibitor JQ1: implications for individualized medicine efforts
Source: EMBO Mol Med. 2017 Mar 8;9(4):482–97. doi: 10.15252/emmm.201606975 (PMC5376755; doi:10.15252/emmm.201606975)
Supplement: Supplementary file 1 — Appendix [file EMMM-9-482-s001.pdf]

Appendix

Bian et. al. (EMM-2016-06975)

Table of content :

-Appendix Figure S1
-Appendix Figure S2
-Appendix Table S1
-Appendix Table S2

Appendix Figure S1. Microphotography showing the different Ki67 staining levels in panel A and differentiation degrees in panel B used for the semi quantitative scoring. For panel A, score 0 [no tumor nuclei positive staining]; score 1 [1-25% tumor nuclei positive staining]; score 2 [26-50% tumor nuclei positive staining]; score 3 [51-75% tumor nuclei staining]; score 4 [76-100% tumor nuclei positive staining]. The percentage of tumor cell staining was independently counted by three authors (BB, MB, and ND) and with « eyeballing » methodology. For panel B, score 0 correspond to the fully undifferentiated state, score 1 to the moderately differentiated state and score 2 correspond to the fully differentiated state. The differentiation degrees were independently appreciated by two independent pathologists (V.S and S.G.). Scale bars represent 100 µm in both panels.

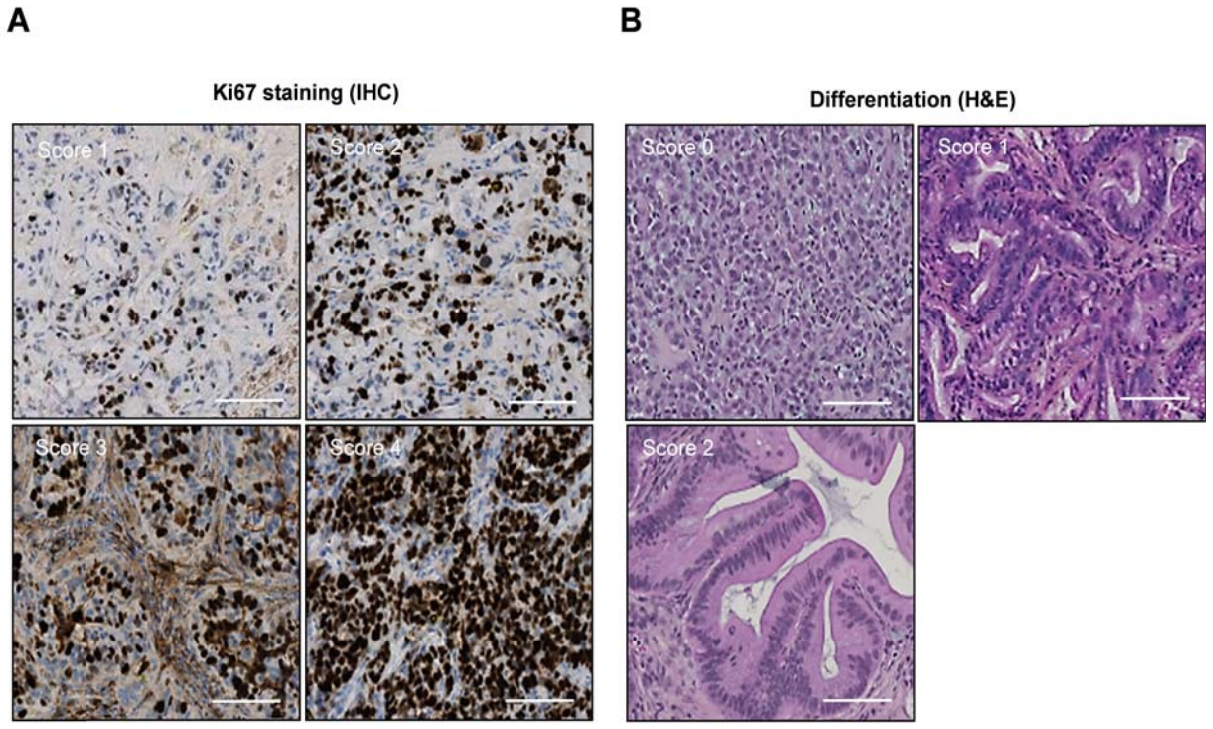

Figure S1

Appendix Figure S2: c-Myc copy number analysis in MYC-high and MYC-low subgroups.

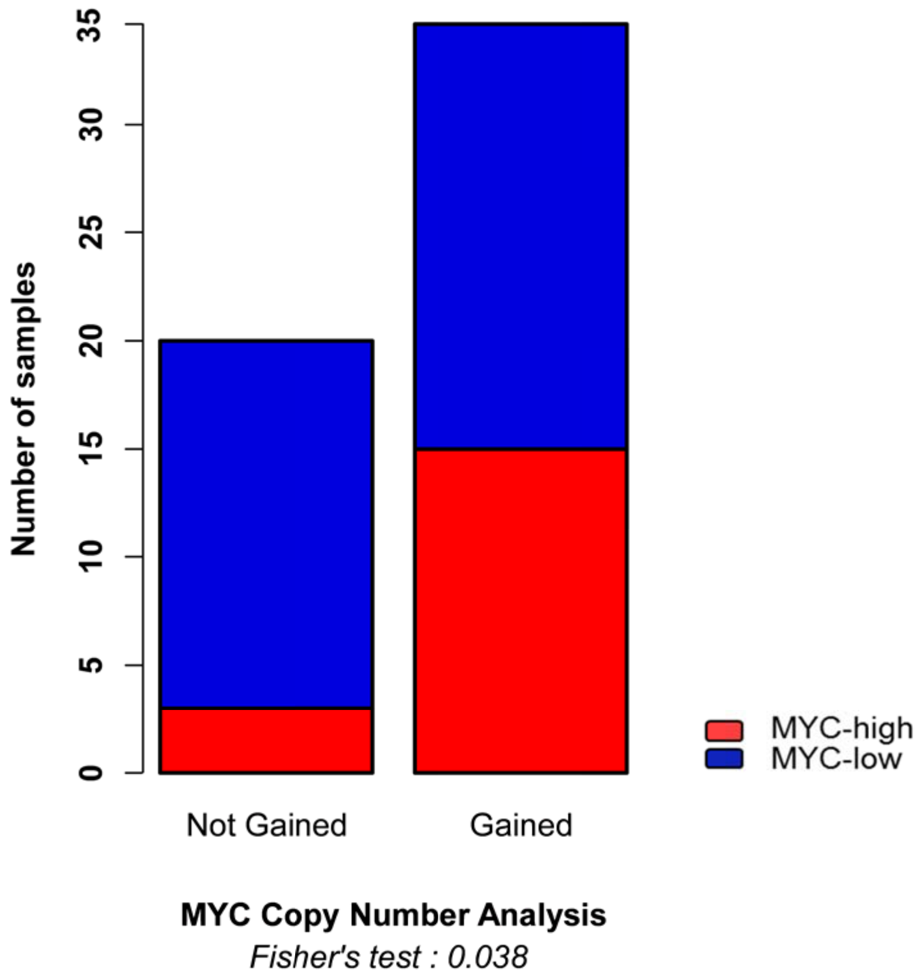

Figure S2

SNP arrays analysis Illumina Infinium HumanCode - BeadChip SNP arrays were used to analyze the DNA samples. Data is expressed as *MYC* gained (one or more alleles) or not gained in MYC-high and MYC-low samples.

Appendix Table S1: List of the 239 c-MYC target transcripts used for differential expression analysis between MYC-high and MYC-low subgroups.

Rank	gene symbol	Affymetrix probeset ID's	T-Test (median)	p-value	FDR(BH)	Fold Change
1	**KPNA2**	16837270	8,98	0.001996	0.004458	1,74
2	**SNRPD2**	16873391	7,76	0.001996	0.004458	1,31
3	**YWHAQ**	16894361	7,64	0.001996	0.004458	1,2
4	**RUVBL2**	16863946	7,56	0.001996	0.004458	1,48
5	**PLK1**	16817017	7,03	0.001996	0.004458	1,6
6	**ERH**	16794256	6,84	0.001996	0.004458	1,36
7	**MCM2**	16945101	6,7	0.001996	0.004458	1,51
8	**CCNA2**	16979515	6,6	0.001996	0.004458	1,61
9	**CDC20**	16663514	6,58	0.001996	0.004458	1,73
10	**XPO1**	16898110	6,57	0.001996	0.004458	1,33
11	**PSMA2**	17057035	6,5	0.001996	0.004458	1,34
12	**TCOF1**	16991078	6,31	0.001996	0.004458	1,37
13	**PSMD7**	16821021	6,22	0.001996	0.004458	1,4
14	**RFC4**	16962493	6,21	0.001996	0.004458	1,65
15	**EIF4H**	17047073	6	0.001996	0.004458	1,28
16	**KARS**	16828514	5,95	0.001996	0.004458	1,38
17	**MAD2L1**	16979389	5,75	0.001996	0.004458	1,68
18	**PHB2**	16760649	5,7	0.001996	0.004458	1,56
19	**CCT4**	16898175	5,6	0.001996	0.004458	1,42
20	**CDK4**	16766683	5,53	0.001996	0.004458	1,58
21	**PSMD14**	16886919	5,49	0.001996	0.004458	1,33
22	**TRIM28**	16866337	5,44	0.001996	0.004458	1,36
23	**HSPD1**	16906962	5,43	0.001996	0.004458	1,39
24	**MCM7**	17060412	5,31	0.001996	0.004458	1,55
25	**PLK4**	16970563	5,26	0.001996	0.004458	1,95
26	**C1QBP**	16840284	5,04	0.001996	0.004458	1,34
27	**MCM4**	17068782	5,02	0.001996	0.004458	1,61
28	**CCT5**	16983172	4,98	0.001996	0.004458	1,37
29	**PRDX4**	17102111	4,97	0.001996	0.004458	1,31
30	**MRTO4**	16660199	4,93	0.001996	0.004458	1,36
31	**PRPS2**	17101517	4,92	0.001996	0.004458	1,36

32	**NDUFAB1**	16825097	4,92	0.001996	0.004458	1,32
33	**TBRG4**	17057433	4,83	0.001996	0.004458	1,21
34	**PRPF31**	16865193	4,77	0.001996	0.004458	1,28
35	**RRM1**	16721126	4,74	0.001996	0.004458	1,47
36	**EIF2S2**	16918485	4,69	0.001996	0.004458	1,29
37	**MCM6**	16903090	4,68	0.001996	0.004458	1,55
38	**PCNA**	16916958	4,61	0.001996	0.004458	1,44
39	**SSB**	16887334	4,58	0.001996	0.004458	1,25
40	**SRM**	16681611	4,54	0.001996	0.004458	1,7
41	**PRDX3**	16718922	4,54	0.001996	0.004458	1,42
42	**LAS1L**	17111688	4,5	0.001996	0.004458	1,33
43	**HNRNPR**	16683271	4,44	0.001996	0.004458	1,25
44	**EIF1AX**	17109706	4,36	0.001996	0.004458	1,37
45	**GSPT1**	16824004	4,35	0.001996	0.004458	1,34
46	**KPNB1**	16835272	4,28	0.001996	0.004458	1,23
47	**PPM1G**	16895848	4,27	0.001996	0.004458	1,29
48	**EIF3M**	16723294	4,24	0.001996	0.004458	1,29
49	**CANX**	16993397	4,23	0.003992	0.007633	1,15
50	**PSMA1**	16736049	4,21	0.001996	0.004458	1,25
51	**RNPS1**	16823097	4,14	0.001996	0.004458	1,25
52	**PTGES3**	16766283	4,14	0.001996	0.004458	1,21
53	**RPL6**	16770445	4,12	0.001996	0.004458	1,33
54	**H2AFZ**	16978334	4,06	0.001996	0.004458	1,29
55	**UTP20**	16755750	4,05	0.001996	0.004458	1,71
56	**TRA2B**	16962359	4,05	0.007984	0.01344	1,18
57	**RANBP1**	16927198	4,02	0.003992	0.007633	1,24
58	**TYMS**	16850477	4,01	0.001996	0.004458	1,7
59	**NHP2**	17003479	4,01	0.001996	0.004458	1,4
60	**ETF1**	17000465	4	0.001996	0.004458	1,31
61	**USP1**	16665447	3,99	0.001996	0.004458	1,39
62	**CNBP**	16958953	3,93	0.001996	0.004458	1,18
63	**GLO1**	17018812	3,9	0.003992	0.007633	1,35
64	**PSMA7**	16920910	3,9	0.001996	0.004458	1,32
65	**RAN**	16759202	3,84	0.001996	0.004458	1,45
66	**VDAC3**	17068541	3,82	0.001996	0.004458	1,31
67	**SF3B3**	16820787	3,81	0.001996	0.004458	1,36

68	**STARD7**	16900450	3,8	0.001996	0.004458	1,26
69	**PABPC4**	16685669	3,79	0.001996	0.004458	1,27
70	**CDK2**	16752305	3,76	0.001996	0.004458	1,31
71	**GOT2**	16826985	3,71	0.001996	0.004458	1,46
72	**SNRPB2**	16911605	3,71	0.001996	0.004458	1,26
73	**SSBP1**	17052394	3,68	0.01198	0.01947	1,18
74	**PSMB2**	16685144	3,67	0.001996	0.004458	1,22
75	**DHX15**	16974873	3,65	0.005988	0.0106	1,13
76	**SRSF1**	16846993	3,65	0.007984	0.01344	1,15
77	**CAD**	16878137	3,63	0.001996	0.004458	1,51
78	**TCP1**	17025297	3,62	0.001996	0.004458	1,45
79	**NOC4L**	16759500	3,62	0.001996	0.004458	1,34
80	**G3BP1**	16991261	3,61	0.001996	0.004458	1,21
81	**CCT7**	16881485	3,59	0.001996	0.004458	1,33
82	**NOP16**	17003050	3,57	0.001996	0.004458	1,35
83	**DDX21**	16705474	3,57	0.001996	0.004458	1,56
84	**CCT2**	16753964	3,54	0.001996	0.004458	1,41
85	**VDAC1**	16999985	3,54	0.001996	0.004458	1,22
86	**CLNS1A**	16742491	3,53	0.001996	0.004458	1,23
87	**MYBBP1A**	16840018	3,51	0.005988	0.0106	1,27
88	**SUPV3L1**	16705531	3,51	0.001996	0.004458	1,75
89	**PGK1**	17105047	3,49	0.003992	0.007633	1,18
90	**WDR74**	16739608	3,48	0.001996	0.004458	1,44
91	**RPLP0**	16771216	3,47	0.005988	0.0106	1,28
92	**TXNL4A**	16856153	3,44	0.003992	0.007633	1,55
93	**PSMC6**	16784228	3,42	0.005988	0.0106	1,17
94	**GNB2L1**	17004092	3,41	0.001996	0.004458	1,22
95	**RABEPK**	17089037	3,39	0.001996	0.004458	1,23
96	**EIF2S1**	16785631	3,37	0.003992	0.007633	1,43
97	**WDR43**	16878556	3,36	0.001996	0.004458	1,44
98	**DCTPP1**	16825794	3,28	0.001996	0.004458	1,36
99	**SNRPG**	16898764	3,27	0.001996	0.004458	1,33
100	**MCM5**	16929573	3,26	0.003992	0.007633	1,43
101	**UNG**	16756627	3,24	0.001996	0.004458	1,42
102	**HSP90AB1**	17009217	3,23	0.003992	0.007633	1,13
103	**EIF3B**	17042946	3,22	0.003992	0.007633	1,28

104	**PSMA6**	16783389	3,16	0.003992	0.007633	1,27
105	**PSMA4**	16803540	3,16	0.03792	0.0546	1,18
106	**NIP7**	16820584	3,15	0.001996	0.004458	1,42
107	**NOP56**	16910825	3,15	0.001996	0.004458	1,46
108	**NDUFAF4**	17021837	3,11	0.003992	0.007633	1,44
109	**XRCC6**	16930678	3,06	0.02595	0.039	1,18
110	**VBP1**	17108562	3,04	0.001996	0.004458	1,22
111	**SNRPA**	16862333	3,03	0.003992	0.007633	1,36
112	**TOMM70A**	16956773	3,03	0.005988	0.0106	1,19
113	**TUFM**	16825410	3	0.001996	0.004458	1,26
114	**ABCE1**	16971139	2,97	0.003992	0.007633	1,47
115	**IARS**	17095775	2,97	0.001996	0.004458	1,32
116	**PA2G4**	16752453	2,95	0.003992	0.007633	1,24
117	**NPM1**	16992324	2,94	0.001996	0.004458	1,37
118	**SNRPA1**	16813900	2,94	0.02595	0.039	1,14
119	**YWHAE**	16839331	2,91	0.001996	0.004458	1,17
120	**PUS1**	16759395	2,9	0.001996	0.004458	1,18
121	**ODC1**	16894402	2,9	0.005988	0.0106	1,82
122	**FBL**	16872267	2,89	0.001996	0.004458	1,48
123	**SRSF2**	16849321	2,88	0.005988	0.0106	1,16
124	**NAP1L1**	16767756	2,87	0.001996	0.004458	2,12
125	**NOP2**	16760437	2,87	0.01198	0.01947	1,43
126	**POLD2**	17057174	2,86	0.001996	0.004458	1,27
127	**TFDP1**	16776905	2,85	0.06387	0.08674	1,11
128	**LSM14B**	16915570	2,83	0.007984	0.01344	1,15
129	**SET**	17089798	2,83	0.007984	0.01344	1,25
130	**FARSA**	16869372	2,83	0.001996	0.004458	1,3
131	**UBA2**	16860746	2,8	0.001996	0.004458	1,23
132	**EXOSC5**	16872567	2,78	0.003992	0.007633	1,16
133	**CDC45**	16927052	2,77	0.003992	0.007633	1,21
134	**IMP4**	16885526	2,76	0.001996	0.004458	1,26
135	**RPS3**	16729103	2,76	0.005988	0.0106	1,21
136	**XPOT**	16753465	2,72	0.001996	0.004458	1,66
137	**HDAC2**	17022854	2,71	0.003992	0.007633	1,19
138	**RPS5**	16866282	2,71	0.01198	0.01947	1,2
139	**PHB**	16846416	2,7	0.02994	0.04417	1,12

140	**SLC25A3**	16755520	2,7	0.001996	0.004458	1,33
141	**CBX3**	17044423	2,7	0.007984	0.01344	1,2
142	**LDHA**	16722603	2,7	0.04192	0.05963	1,17
143	**IPO4**	16791086	2,69	0.001996	0.004458	1,31
144	**RPL14**	16939495	2,67	0.07585	0.09906	1,13
145	**DUT**	16800839	2,66	0.03593	0.05204	1,18
146	**ACP1**	16876440	2,65	0.01996	0.03098	1,13
147	**HNRNPA1**	16751993	2,64	0.03992	0.05713	1,2
148	**AIMP2**	17043355	2,61	0.005988	0.0106	1,19
149	**SNRPD1**	16851344	2,57	0.007984	0.01344	1,47
150	**PSMC4**	16862145	2,47	0.01397	0.02226	1,23
151	**APEX1**	16781516	2,45	0.003992	0.007633	1,31
152	**PWP1**	16756475	2,44	0.001996	0.004458	1,59
153	**BYSL**	17008544	2,42	0.02395	0.0367	1,17
154	**RPL18**	16873987	2,42	0.00998	0.01656	1,12
155	**EEF1B2**	16889938	2,41	0.02794	0.04148	1,2
156	**GRWD1**	16863783	2,38	0.02794	0.04148	1,1
157	**IFRD1**	17050381	2,34	0.01597	0.02527	1,24
158	**SRSF7**	16896650	2,33	0.06387	0.08674	1,13
159	**COX5A**	16811739	2,33	0.007984	0.01344	1,14
160	**BUB3**	16710271	2,31	0.01796	0.02806	1,38
161	**COPS5**	17077888	2,3	0.02196	0.03385	1,15
162	**RRP12**	16717106	2,28	0.001996	0.004458	1,31
163	**POLE3**	17097495	2,27	0.05389	0.07532	1,12
164	**SF3A1**	16933818	2,26	0.06786	0.09061	1,1
165	**EIF3D**	16934583	2,23	0.08583	0.1109	1,13
166	**HNRNPU**	16701324	2,22	0.03194	0.04683	1,12
167	**PSMD3**	16833934	2,22	0.005988	0.0106	1,3
168	**HDDC2**	17023308	2,19	0.09182	0.1161	1,27
169	**RSL1D1**	16823991	2,16	0.02595	0.039	1,41
170	**AP3S1**	16988174	2,15	0.001996	0.004458	1,26
171	**SRPK1**	17018530	2,08	0.0499	0.07015	1,06
172	**RRP9**	16954623	2,07	0.09381	0.1174	1,1
173	**PSMD8**	16861704	2,01	0.07585	0.09906	1,14
174	**PCBP1**	16881153	2,01	0.1557	0.1833	1,12
175	**DDX18**	16884877	2	0.01796	0.02806	1,2

176	**ORC2**	16907173	1,96	0.07385	0.09752	1,17
177	**ILF2**	16693515	1,86	0.1078	0.1321	1,11
178	**CSTF2**	17105347	1,86	0.03593	0.05204	1,16
179	**PPIA**	17045738	1,85	0.1098	0.1339	1,22
180	**RAD23B**	17087933	1,84	0.07385	0.09752	1,18
181	**MRPL9**	16693173	1,84	0.05988	0.08225	1,2
182	**CUL1**	17053019	1,83	0.01397	0.02226	1,23
183	**IMPDH2**	16953813	1,8	0.04591	0.06492	1,3
184	**NMD3**	16947626	1,75	0.08383	0.1089	1,23
185	**SYNCRIP**	17021385	1,71	0.1357	0.1614	1,12
186	**EIF4E**	16978142	1,68	0.1597	0.1871	1,09
187	**CCT3**	16694529	1,66	0.09381	0.1174	1,07
188	**SRSF3**	17007996	1,63	0.05788	0.07997	1,06
189	**EXOSC7**	16940036	1,58	0.1697	0.1978	1,15
190	**PSMB3**	16833698	1,57	0.1178	0.1422	1,21
191	**RPL22**	16681064	1,54	0.1058	0.1303	1,09
192	**PRMT3**	16722799	1,53	0.06786	0.09061	1,39
193	**RCL1**	17083295	1,53	0.0978	0.1217	1,15
194	**CYC1**	17073565	1,5	0.01397	0.02226	1,29
195	**CTNS**	16829738	1,48	0.1018	0.1261	1,12
196	**NOLC1**	16708552	1,43	0.08782	0.1122	1,51
197	**PPRC1**	16708533	1,4	0.06587	0.08894	1,31
198	**UBE2E1**	16938344	1,38	0.2754	0.3105	1,06
199	**EIF3J**	16800431	1,38	0.4092	0.4445	1,12
200	**TARDBP**	16658926	1,33	0.00998	0.01656	1,31
201	**RPS6**	17092737	1,28	0.1717	0.1992	1,17
202	**MYC**	17072669	1,24	0.1138	0.138	1,4
203	**EIF4G2**	16735801	1,24	0.3852	0.4204	1,01
204	**HK2**	16881838	1,22	0.4331	0.4642	1,04
205	**HNRNPD**	16977340	1,18	0.6108	0.6402	1,02
206	**MPHOSPH10**	16881274	1,17	0.08782	0.1122	1,15
207	**HPRT1**	17107045	1,16	0.3214	0.3572	1,16
208	**NCBP2**	16963402	1,15	0.1277	0.1534	1,15
209	**U2AF1**	16926148	1,15	0.3713	0.4089	1,09
210	**DEK**	17015987	1,14	0.2236	0.2569	1,13
211	**EPRS**	16699392	1,13	0.3832	0.4202	1,05

212	**TFB2M**	16701407	1,11	0.2635	0.2984	1,05
213	**PES1**	16933931	1,07	0.1756	0.2028	1,19
214	**GNL3**	16941661	1,04	0.5349	0.5657	1,11
215	**PSMD1**	16892039	1,03	0.6906	0.7145	1,04
216	**HNRNPA2B1**	17056031	0.9459	0.1437	0.17	1,06
217	**SNRPD3**	16928282	0.8702	0.3074	0.3433	1,05
218	**SMARCC1**	16953199	0.8071	0.2275	0.2602	1,01
219	**MRPL23**	16720820	0.8037	0.3453	0.3821	1,06
220	**HDGF**	16694742	0.7125	0.4132	0.4448	1,02
221	**SERBP1**	16688308	0.5663	0.6467	0.6749	1,06
222	**MAP3K6**	16683960	0.558	0.2834	0.318	1,03
223	**LSM7**	16866940	0.5212	0.6647	0.6907	1,05
224	**SLC29A2**	16740778	0.4233	0.5349	0.5657	1,01
225	**UBE2L3**	16927615	0.3884	0.7505	0.7731	1,03
226	**RPS19BP1**	16935264	0.3745	0.9321	0.9321	1,02
227	**TMEM97**	16832429	0.3107	0.8723	0.8833	1,04
228	**RPL34**	16969624	0.3099	0.9142	0.918	1,02
229	**NCBP1**	17087343	0.2708	0.7745	0.791	1,08
230	**HNRNPA3**	16888031	-0.2364	0.8244	0.8384	-1,08
231	**MRPS18A**	17019516	-0.2544	0.8902	0.8977	-1,01
232	**RPRML**	16846035	-0.4361	0.5788	0.6094	-1,04
233	**PABPC1**	17079672	-0.4857	0.7605	0.7801	1,12
234	**SLC19A1**	16926595	-0.5267	0.525	0.5601	-1,02
235	**HNRNPC**	16790233	-1,3	0.1357	0.1614	-1,1
236	**PPA2**	16978661	-1,32	0.4132	0.4448	-1,03
237	**DUSP2**	16900441	-1,83	0.2335	0.2658	-1,07
238	**SORD**	16800506	-1,94	0.09182	0.1161	-1,2
239	**FAM120A**	17086921	-2,12	0.05788	0.07997	-1,11

Table S2. List of primers used for amplification of the sixteen markers.

Transcripts	Forward primer sequence (5'-3')	F primers position	Reverse primer sequence (5'-3')	R primers position
**CDC20**	GCACAGTTCGCGTTCGAGA	4-22	CTGGATTTGCCAGGAGTTCGG	191-171
**KPNA2**	CTGCCCGTCTTCACAGATTCA	32-52	GCGGAGAAGTAGCATCATCAGG	190-169
**PLK1**	AAAGAGATCCCGGAGGTCCTA	112-132	GGCTGCGGTGAATGGATATTTC	322-301
**SRM**	GTGGTGGCCTATGCCTACTG	688-707	CTCCTGGAAGTTCGTGCTCG	783-764
**RFC4**	CCGCTGACCAAGGATCGAG	43-61	AGGGAACGGGTTTGGCTTTC	118-99
**MCM2**	ATGGCGGAATCATCGGAATCC	1-21	GGTGAGGGCATCAGTACGC	117-99
**RUVBL2**	GTCGGGCAGTCCTTATTGCT	209-228	TGGTCGATCAATCTGGATCTCC	450-429
**MAD2L1**	GTTCTTCTCATTCGGCATCAACA	66-88	GAGTCCGTATTTCTGCACTCG	153-133
**CCT4**	ATGCCCGAGAATGTGGCAC	1-19	GCTTGTCGCGGTCCTGATAG	88-69
**CAD**	AGTGGTGTTTCAAACCGGCAT	78-98	CAGAGGATAGGTGAGCACTAAGA	168-146
**VSIG2**	CATCTCTGA GTCCCATCCAATCC	201-223	TGACCCGCTTTGACTTAGAAC	277-257
**BCL2L15**	TTGCCAGCCGGAACCTATG	86-104	CGAAGGCGACCAGCAATGATA	173-153
**RAB25**	ATCGGCGAATCAGGTGTGG	52-70	TAGGTCTGGTGCTTGGTTAGG	299-279
**TXNIP**	GGTCTTTAACGACCCTGAAAAGG	36-58	ACACGAGTAACTTCACACACCT	122-101
**CTSE**	AGGCATCCGTCCCTCAAGAA	82-101	CCTTGGCACTCTGGTCCATTG	202-182
**ERN2**	GAGTCCACAGGTTCATACTCTCA	228-250	CTGTCTGCTTGCTTAGTGCG	316-297
**28S subunit**	AACGAGATTCCCACTGTCCC	3824-3843	CCGTAAAACGACGGCCAG	3943-3925
