# Supplementary material for: Gene expression profiling of patient‐derived pancreatic cancer xenografts predicts sensitivity to the BET bromodomain inhibitor JQ1: implications for individualized medicine efforts
Source: EMBO Mol Med. 2017 Mar 8;9(4):482–97. doi: 10.15252/emmm.201606975 (PMC5376755; doi:10.15252/emmm.201606975)
Supplement: Supplementary file 8 — Source Data for Appendix [file EMMM-9-482-s008.zip › emmm201606975-sup-0014-SDataAppendix.pdf]

sampleName	rs7462301	rs1268004[illegible]	MYC_status
PDAC028T	1	1	1
PDAC003T	0	0	0
PDAC004T	1	1	1
PDAC013T	1	1	1
PDAC014T	1	1	1
PDAC031T	1	1	1
PDAC001T	1	1	1
PDAC021T	1	1	1
PDAC087T	2	2	2
PDAC089T	1	1	1
PDAC091T	1	1	1
PDAC053T	1	1	1
PDAC078T	0	0	0
PDAC068T	1	1	1
PDAC007T	-1	-1	-1
PDAC054T	1	1	1
PDAC022T	1	1	1
PDAC016T	1	1	1
PDAC011T	0	0	0
PDAC019T	0	0	0
PDAC032T	0	0	0
PDAC025T	0	0	0
PDAC018T	1	1	1
PDAC029T	0	0	0
PDAC030T	1	1	1
PDAC009T	2	2	2
PDAC024T	0	0	0
PDAC027T	2	2	2
PDAC020T	0	0	0
PDAC012T	1	1	1
PDAC017T	1	1	1
PDAC008T	0	0	0
PDAC015T	1	1	1
PDAC006T	1	1	1
PDAC026T	1	1	1
PDAC037T	1	1	1
PDAC086T	0	0	0
PDAC036T	2	2	2
PDAC069T	0	0	0
PDAC043T	0	0	0
PDAC079T	1	1	1
PDAC085T	0	0	0
PDAC082T	2	2	2
PDAC083T	0	0	0
PDAC081T	1	1	1
PDAC070T	1	1	1
PDAC056T	1	1	1
PDAC058T	0	0	0
PDAC057T	1	1	1
PDAC076T	0	0	0
PDAC074T	0	0	0
PDAC041T	1	1	1
PDAC084T	1	1	1
PDAC088T	0	0	0

	IlmnID	id	IlmnStrand	SNP	AddressA_ID	AlleleA_ProbeSeq	AddressB_ID	AlleleB_ProbeS	GenomeBuild	chrom	loc	Ploidy	Species	Source	SourceVersion	SourceStra	SourceSeq	TopGenomicSeq	BeadSetID	Exp_Clusters	RefStrand	chrNum	highConf
rs7462301	rs7462301	rs7462301	TOP	[A/G]	89653494	CAATACTGAGGTTTAGGCAAGTTAAATGGCTGTCCCCAAGTCACACAAGG	#N/A		37,1	8	128757350	diploid	Homo sapiens	dbSNP	131	TOP	TTGAATGTGACAATACTGAGGTTTAGGCAAGTTAAATGGCTGTCCCCAAGTCACACAAGG[A/G]CCAACAACAAAACAACAGTAATATGGCTGAGCGTGGTGGCTCACACTTGTAATCCCAGCA	TTGAATGTGACAATACTGAGGTTTAGGCAAGTTAAATGGCTGTCCCCAAGTCACACAAGG[A/G]CCAACAACAAAACAACAGTAATATGGCTGAGCGTGGTGGCTCACACTTGTAATCCCAGCA	705	3	+	8	0
rs1268004	rs1268004	rs1268004	BOT	[T/C]	57740917	TTCTCCAAGAGTTCCCAAAGCATGATTCCAGACAAGCAGCATCCACATCA	#N/A		37,1	8	128758861	diploid	Homo sapiens	dbSNP	131	BOT	CTGCCCTACCTTCTCCAAGAGTTCCCAAAGCATGATTCCAGACAAGCAGCATCCACATCA[T/C]GTGTGTATTTGCTGCAAATGCGGGTACACAGCCTGAATTAGAATCTCAGGAGATGGGGCC	GGCCCCATCTCCTGAGATTCTAATTCAGGCTGTGTACCCGCATTTGCAGCAAATACACAC[A/G]TGATGTGGATGCTGCTTGTCTGGAATCATGCTTTGGGAACTCTTGGAGAAGGTAGGGCAG	705	3	+	8	0
