# Supplementary material for: Gene expression profiling of patient‐derived pancreatic cancer xenografts predicts sensitivity to the BET bromodomain inhibitor JQ1: implications for individualized medicine efforts
Source: EMBO Mol Med. 2017 Mar 8;9(4):482–97. doi: 10.15252/emmm.201606975 (PMC5376755; doi:10.15252/emmm.201606975)
Supplement: Supplementary file 10 — Source Data for Figure 1 [file EMMM-9-482-s009.pdf]

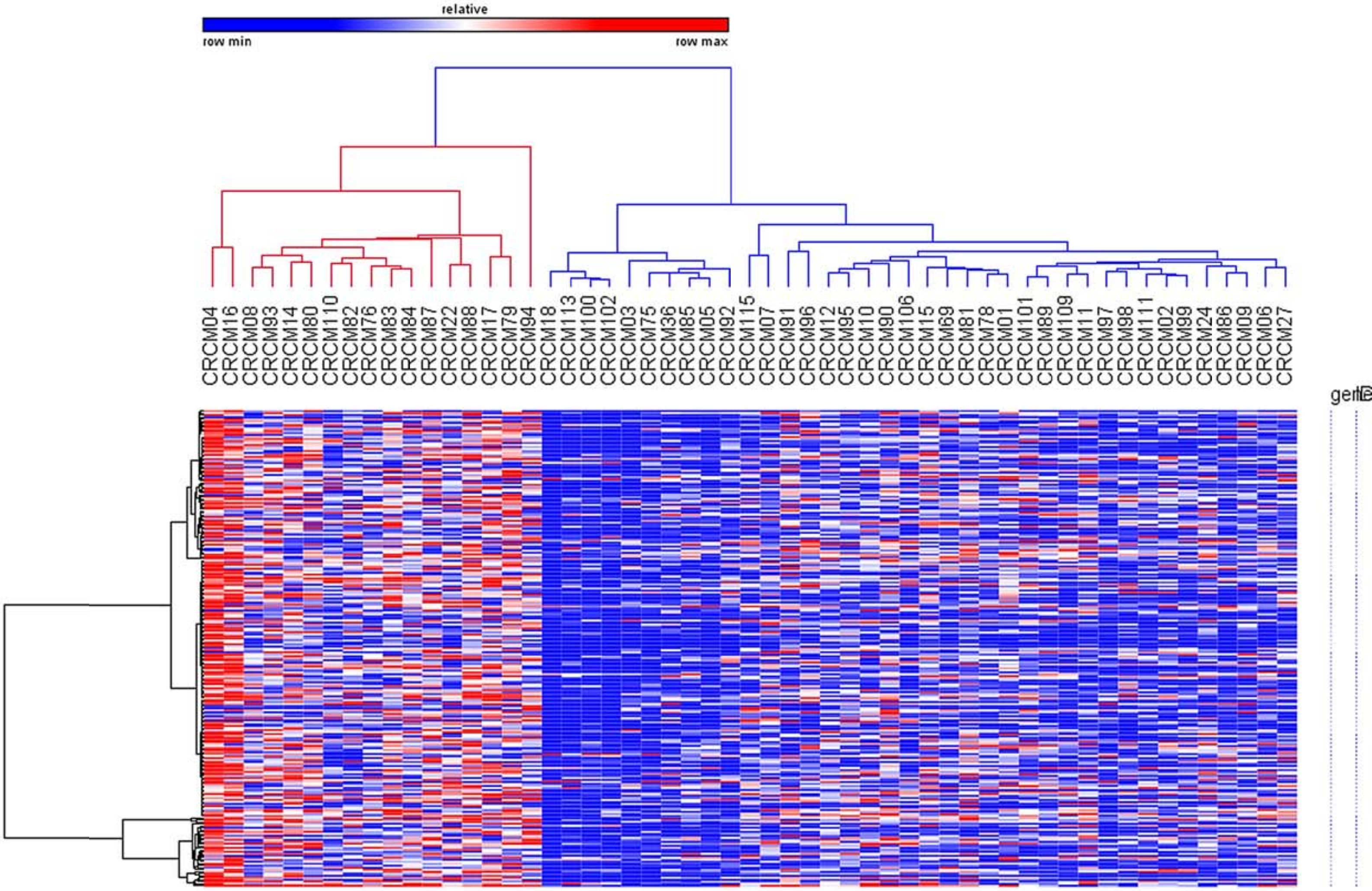

1. Import File

File:	Liste marqueurs MYC V1et V2 formatter pour GSEA.txt

2. Hierarchical Clustering

Cluster columns:	true
Column distance metric:	Euclidean distance
Cluster selected columns only:	false
Cluster columns in space of selected rows only:	false
Group columns:	false
Group columns by:	
Cluster rows:	true
Row distance metric:	Euclidean distance
Cluster selected rows only:	false
Cluster rows in space of selected columns only:	false
Group rows:	false
Group rows by:	
Linkage method:	Complete Linkage

GENE-E 3.0.204

Built 09/02/2015 12:30 PM

figure 1C

ki67 scoring

MYC high	MY Low
3	3,5
1	2
4	2
4	2
2,25	3
2	2
4	3
3	1
2,5	3,5
4	2
4	3
3,5	2
2	2
2	3
4	1,25
2,75	2
1	0,5
	1,75
	2
	2
	2
	1
	2
	2,5
	2
	2
	2,75
	1
	1
	3
	1,5
	3
	2
	3
	2
	1,75
	1
	1,25

differenciation scoring

MYC high	MYC low
2	2
1	2
0	2
0	2
1	2
0	2
1	2
1	1
0	2
1	1
1	2
0	2
2	2
	2
	2
	2
	1
	2
	2
	1
	2
	2

OS kaplan meier

month	MYC high	MYC low		
3,7	1			1=dead
22,75	1			0=alive or lost
5,28	1			
32,46	0			
34,95	0			
6,07	1			
28,13	1			
9,25	1			
3,44	1			
2,79	1			
8,75	1			
18,07	1			
14,52	1			
1,21	1			
6,98	1			
12,26	1			
9,87	1			
29,02		1		
34		1		
25,74		0		
15,28		1		
33,11		0		
11,41		1		
33,21		0		
23,44		1		
23,93		1		
5,44		1		
9,8		1		
14,07		1		
38,82		0		
13,08		1		
38,2		0		
16,79		1		
20,72		1		
20,16		1		
13,48		1		
35,61		0		
3,28		1		
11,51		0		
18,82		1		
10,62		1		
12,66		1		
23,87		0		
9,67		1		
10,39		1		
23,67		0		
17,25		1		

6,79		1
9,57		1
20,82		0
14		0
19,08		0
12,56		1
17,84		0
10,49		1

RFS kaplan meier

month	MYC high	MYC low
3,7	1	
8	1	
4,23	1	
19,93	0	
34,95	0	
5,28	1	
13,08	1	
5,67	1	
3,44	1	
2,79	1	
2,13	1	
18,07	1	
10,13	1	
1,21	1	
6,52	1	
4,36	1	
7,28	1	
11,41		1
21,57		1
14,69		0
7,25		1
33,11		0
8,89		1
12,56		0
11,93		1
13,54		1
3,67		1
9,8		1
9,21		1
38,82		0
5,05		1
38,2		0
10,66		1
16,39		1
20,16		1
13,48		1

16,49	0
3,28	1
11,51	0
2,46	1
10,62	1
12,66	1
23,87	0
7,54	1
10,39	1
5,61	0
8,72	1
4,16	1
7,8	1
20,82	0
11,48	0
16,56	0
5,64	1
12,62	0
10,49	1
