# Supplementary material for: Gene expression profiling of patient‐derived pancreatic cancer xenografts predicts sensitivity to the BET bromodomain inhibitor JQ1: implications for individualized medicine efforts
Source: EMBO Mol Med. 2017 Mar 8;9(4):482–97. doi: 10.15252/emmm.201606975 (PMC5376755; doi:10.15252/emmm.201606975)
Supplement: Supplementary file 12 — Source Data for Figure 3 [file EMMM-9-482-s011.pdf]

figure 3 A		figure 3B		figure 3C	
MYC high	MYC low	MYC high	MYC low	MYC high	MYC low
13,05271	0,5175914	980682,5	2,992186	4227,469	6,198818
10,93506	0,4203209	113775,7	0,7687295	163,419	3,084278
17,15903	0,3251464	405655600	1,842301	741099,8	0,8614749
9,236097	0,4142451	219,3232	1,984856	3,642694	0,6891637
86,83639	0,3833082	488662,8	0,9179869	16315150	10,15133
15,24046	0,4690201	15316,58	0,7165487	5227,288	4,814575
7,905078	0,5323244	432957,6	2,673518	202,0685	2,395535
6,622576	0,4322852	50230,36	0,6868598	916373,9	0,669101
10,39198	0,3344015	179091300	1,646096	4,504211	0,5352682
5,593635	0,4260364	96,82813	1,773469	20173770	7,884461
0,4697425	0,9972171	215737,8	0,8202214	9477,642	3,94201
9,230042	0,4823706	6762,055	0,6402363	366,3722	1,961383
10,7226	0,4810465	582116,9	1,647776	1661486	0,5478371
8,982989	0,3906439	67535,35	0,4233341	8,166625	0,4382594
14,09588	0,3021892	240790400	1,014542	36577250	6,455529
7,587313	0,3849971	130,1866	1,093047	3284,693	6,733052
71,33477	0,3562445	290062,1	0,5055291	126,9746	3,350091
12,51981	0,4359047	9091,666	0,3945985	575825,8	0,9357195
15,09759	0,4905366	174166	1,466333	2,83033	0,748558
12,64819	0,3983505	20206,19	0,376719	12676680	11,02621
19,84722	0,3081508	72043120	0,9028268	5476,036	6,3798
10,68306	0,3925923	38,95109	0,9726868	211,6842	3,174327
100,4405	0,3632724	86784,92	0,4498632	959980,9	0,8866266
17,62808	0,4445041	2720,175	0,3511476	4,718551	0,7092847
12,28466	0,4852033	795109,4	2,721641	21133770	10,44771
10,29163	0,3940195	92246,05	0,6992233	9826,709	2,133704
16,14936	0,3048005	328894000	1,675725	379,8659	1,061644
8,69263	0,3883238	177,821	1,805391	1722679	0,2965295
81,72679	0,3593228	396193,8	0,8349853	8,467406	0,237218
14,34369	0,4396713	12418,24	0,6517605	37924410	3,494204
9,596087	0,7134585	326644,7	4,356323	7114,338	3,578227
8,039239	0,5793789	37896,28	1,119193	275,0152	1,780379
12,61497	0,4481884	135115300	2,682205	1247185	0,4972807
6,790193	0,5710039	73,05195	2,889752	6,130231	0,3978152
63,84037	0,5283598	162763,3	1,336497	27456500	5,859789
11,20448	0,6465069	5101,629	1,043223	6489,285	6,507189
9,811168	0,5082124	378268,8	1,679272	250,8529	3,237711
8,219425	0,4127045	43885,54	0,4314258	1137609	0,9043305
12,89771	0,3192545	156469500	1,033934	5,59164	0,7234474
6,942383	0,4067388	84,59734	1,11394	25044220	10,65633
65,27124	0,3763624	188487	0,5151919	6803,745	5,182193
11,45561	0,4605212	5907,909	0,4021409	263,0088	2,578447
12,54537	0,522448	743614,6	3,203609	1192736	0,7201904
10,51004	0,4242648	86271,8	0,823047	5,862601	0,5761389
16,49208	0,3281973	307593300	1,972475	26257820	8,486483
8,877104	0,418132	166,3045	2,125104	4018,118	4,588573
83,46119	0,3869048	370534,6	0,9828507	155,3263	2,283086
14,64809	0,473421	11613,98	0,7671791	704399,4	0,6376925
16,82854	0,53381	683358,1	2,611107	3,462303	0,510142
14,09831	0,4334915	79281,02	0,6708257	15507200	7,514354
22,12271	0,3353347	282668400	1,607669	25,903	0,16853
11,90787	0,4272253	152,8285	1,732069	108,4531	0,1403407
111,956	0,395319	340509,4	0,801074	69,4697	0,2030893
19,64915	0,4837167	10672,88	0,6252905	1,827088	0,2844276
8,873646	0,4663797	362127,9	1,794547	307922,9	11,38851
7,434005	0,3787334	42012,92	0,4610414	34,87073	0,3317571
11,66525	0,2929757	149792800	1,10491	146	0,2762654
6,278993	0,3732587	80,98752	1,190407	93,52042	0,3997882
59,03415	0,3453828	180444,1	0,5505577	2,459635	0,5599055
10,36095	0,4226142	5655,815	0,4297462	414527,2	22,41867
11,28453	0,2308653	3889,376	0,04420153	27,98562	0,2494968
3,567959	0,3197241	2,027866	0,04536492	117,1728	0,2077644
3,26947	0,2981363	7,853938	0,04740702	75,05511	0,3006594
2,596598	0,3163959	0,6253439	0,05184724	1,973988	0,421075
15,24886	0,2793885	43,79098	0,06818217	332680,2	16,85988
5,857327	0,3028193	9,89165	0,06532966	39,33161	0,2717232
11,89111	0,2903038	2692,957	0,2486759	164,6772	0,2262731
3,759746	0,40204	1,40407	0,2552211	105,4841	0,3274436
3,445213	0,3748943	5,437972	0,2667099	2,774286	0,4585864
2,736172	0,397855	0,4329806	0,2916904	467556,1	18,36184
1,337642	1,105974	30,32034	0,38359	49,80606	0,1769544
6,172175	0,380783	6,848859	0,3675419	208,5327	0,1473559
9,663619	0,2548908	4001,508	0,1141122	133,5757	0,2132412
3,055456	0,3529968	2,08633	0,1171156	3,51311	0,2986454
2,799843	0,3291625	8,08037	0,1223876	592071,6	11,95779
2,223622	0,3493223	0,6433728	0,1338506	35,61829	0,1984654
13,05851	0,3084636	45,05349	0,1760214	149,13	0,1652689
5,01598	0,3343329	10,17683	0,1686573	95,52531	0,2391633
8,843377	0,2311905	737,1301	0,9210549	2,512364	0,3349494
2,796111	0,3201743	0,3843292	0,9452974	423413,9	13,41141
2,562194	0,2985562	1,48851	0,9878498	42,3034	0,1458564
2,034882	0,3168415	0,1185177	1,080374	177,1198	0,1214596
11,95011	0,2797819	8,299441	1,420755	113,4542	0,1757662
4,590227	0,3032458	1,874705	1,361315	2,983904	0,2461615
9,735603	0,2243887	2714,152	0,2234059	502883,4	9,856327
3,078216	0,3107546	1,415121	0,229286	31,59212	0,3598412
2,820699	0,2897725	5,480772	0,2396073	132,2728	0,299652
2,240186	0,3075198	0,4363884	0,2620493	84,72745	0,4336314
13,15578	0,2715506	30,55898	0,3446102	2,228375	0,607303
5,053344	0,2943241	6,902764	0,330193	375552,6	24,31647
9,035256	0,2858107	3030,941	0,2689119	34,99581	0,2658637
2,85678	0,3958175	1,58029	0,2759897	146,5237	0,2213937
2,617787	0,369092	6,120473	0,2884133	93,85588	0,3203825
2,079034	0,3916973	0,4873225	0,3154266	2,468457	0,4486974
12,2094	0,3458822	34,12575	0,4148045	416014,2	17,96588
4,689823	0,3748896	7,708436	0,3974506	26,40541	0,3114766
9,751298	0,2002251	3671,518	0,1870013	110,5566	0,2593771
3,083179	0,2772906	1,914277	0,1919232	70,81713	0,375349
2,825246	0,258568	7,41401	0,2005627	1,862526	0,5256782
2,243797	0,2744042	0,5903161	0,2193477	313895,4	21,0482
13,17699	0,2423083	41,33808	0,2884551	125,1307	0,0475658
5,06149	0,2626294	9,337582	0,2763871	15,12452	0,1424616
10,82821	0,2782813	2343,213	0,1762729	1094590	213,9817
3,423677	0,3853901	1,221718	0,1809125	1,79091	0,2423732
3,137259	0,3593686	4,731722	0,1890562	336921,3	0,03296215
2,491596	0,3813784	0,3767478	0,2067636	127,3129	0,07365581
14,63222	0,3367702	26,38253	0,2719062	15,38829	0,2206024
5,620468	0,3650135	5,959372	0,2605306	1113679	331,3515
8,889603	0,2624869	3826,762	0,273836	1,822142	0,3753159
2,810727	0,3635166	1,995219	0,2810435	342797,1	0,05104202
2,575587	0,3389719	7,727499	0,2936946	81,18177	0,07514699
2,045519	0,3597326	0,6152766	0,3212025	9,812428	0,2250685
12,01257	0,3176562	43,08599	0,4224002	710143,8	338,0598
4,61422	0,3442964	9,732406	0,4047285	1,161899	0,3829142
9,957561	0,3102275	4401,729	0,2864339	218586,5	0,05207537
3,148395	0,4296322	2,294999	0,293973	157,9105	0,1144095
2,885007	0,4006234	8,888548	0,3072061	19,08662	0,3426614
2,291259	0,42516	0,7077213	0,3359796	1381334	514,6878
13,45571	0,3754308	49,55962	0,4418329	2,260065	0,5829776
5,168553	0,4069163	11,19469	0,4233481	425182,9	0,07928349
2,978381	0,5445813	1,500809	1,752431	61,44464	0,146334
5,409888	0,3625125	1,339082	1,152558	7,426804	0,4382769
3,471481	0,4016891	0,8702412	0,522576	537491,8	658,3052
4,615306	0,3808991	1,105775	1,246758	0,879415	0,7456505
2,708576	0,3408886	7,2386	0,979638	165443,1	0,1014066
3,151775	0,3502063	2,285743	0,5580487	55,8606	0,1561184
3,439495	0,5653536	2,727526	2,810592	6,751862	0,4675814
6,247448	0,3763401	2,433608	1,848501	488644,9	702,3215
4,008937	0,417011	1,581551	0,8381202	0,7994944	0,795507
5,32985	0,395428	2,009604	1,999581	150407,8	0,108187
1,046231	0,9688901	13,15522	1,571167	74,05939	0,07279321
3,639734	0,3635645	4,154043	0,8950122	8,951547	0,2180189
3,913311	0,5348597	6,783048	1,697958	647840,3	327,4709
7,108081	0,3560411	6,052107	1,116732	1,059961	0,3709204
4,561197	0,3945183	3,933138	0,5063322	199409,1	0,05044426
6,064076	0,3740995	4,997656	1,208004	58,43457	0,08440806
3,558812	0,3348033	32,71555	0,9491869	7,062976	0,2528059
4,141134	0,3439546	10,33063	0,5407023	511160,8	379,7221
2,660764	0,6926266	1,430129	7,226569	0,8363338	0,4301043
4,832973	0,4610622	1,276018	4,752849	157338,3	0,05849312
3,101279	0,5108889	0,8292575	2,154967	134,9937	0,06299049
4,123127	0,4844472	1,053699	5,141304	16,31667	0,1886593
2,419731	0,4335597	6,897701	4,039772	1180868	283,3719
2,815667	0,4454104	2,178097	2,301247	1,932073	0,3209703
4,364975	0,4691666	7,288043	1,675707	363478,2	0,04365116
7,928477	0,3123111	6,502683	1,102097	101,3489	0,09241822
5,087639	0,3460624	4,225958	0,4996967	12,25002	0,2767966
6,763976	0,3281515	5,369729	1,192173	886557,2	415,757
3,969561	0,2936817	35,1512	0,9367477	1,450537	0,4709204
4,619093	0,301709	11,09974	0,5336164	272887,6	0,064044
3,561502	0,6891658	5,649847	1,880671	25,13745	180,2525
6,469061	0,4587584	5,041019	1,2369	0,2868825	149,3537
4,151144	0,5083362	3,276053	0,560817	0,2647129	4,810931
5,518913	0,4820266	4,162729	1,337993	1,033265	7,262496
3,238873	0,4313935	27,24996	1,051326	0,6424957	3954,482
3,768844	0,4431849	8,604757	0,5988856	30,61983	127,3129
4,993539	0,5914846	13,44168	2,113532	0,3494505	105,489
9,070192	0,3937348	11,99321	1,390051	0,3224458	3,397975
5,820268	0,4362855	7,794134	0,6302564	1,258616	5,129523
7,738	0,4137049	9,903645	1,503661	0,7826215	2793,063
4,541185	0,3702485	64,83102	1,181499	31,71186	110,1332
5,28425	0,3803686	20,47178	0,6730385	0,3619134	91,25426
2,5789	0,7129112	2,596796	1,866898	0,3339456	2,939451
4,684277	0,4745651	2,316965	1,227842	1,303504	4,437343
3,005862	0,5258511	1,505747	0,55671	0,8105331	2416,165
3,99627	0,498635	1,913283	1,328195	37,78939	64,84686
2,345283	0,4462573	12,52469	1,043627	0,4312736	53,73085
2,729037	0,458455	3,954939	0,5944998	0,3979458	1,73076
3,287509	0,5835065	6,422287	2,191912	1,553318	2,612724
5,971384	0,3884239	5,730221	1,441601	0,9658707	1422,647
3,831788	0,4304007	3,723951	0,6536294	41,60152	57,00487
5,094332	0,4081247	4,731852	1,559424	0,4747797	47,23313
2,989701	0,3652544	30,97554	1,225315	0,4380898	1,521457
3,478899	0,3752381	9,78119	0,6979981	1,710014	2,296765
2,920595	0,5652823	3,780108	2,074567	1,063306	1250,605
5,304926	0,3762926	3,372764	1,364424	33,71218	141,5792
3,404128	0,4169584	2,191889	0,6186372	0,3847422	117,3098
4,525761	0,3953781	2,785131	1,47594	0,3550102	3,778743
2,656024	0,3538468	18,23197	1,159718	1,385726	5,704323
3,090625	0,3635186	5,757133	0,6606306	0,86166	3106,045
11,48634	0,331731	2574083	0,0652848	17,19928	218,0812
15,35037	0,3766725	72184,73	0,1639791	0,1962877	180,6979
16,27347	0,5321198	900778,9	0,09498988	0,1811191	5,820577
5,529878	0,4589583	81,90389	0,2455012	0,7069696	8,78664
65,63412	0,4375076	1189957	0,06200302	0,4396016	4784,389
14,84509	0,3705556	57336,4	0,08907211	33,37835	55,8633
11,39561	0,2994416	2176863	0,171769	0,3809324	46,28725
15,22911	0,3400087	61045,52	0,4314408	0,3514948	1,490989
16,14493	0,4803253	761774,8	0,2499252	1,372004	2,25077
5,486197	0,4142851	69,26485	0,6459311	0,8531275	1225,561
1,284563	0,7730378	1006328	0,1631344	32,14408	91,85658
14,72783	0,3344872	48488,5	0,2343551	0,3668462	76,11059
11,00823	0,3552043	2201009	0,1780709	0,3384972	2,451648
14,71143	0,4033258	61722,66	0,4472697	1,32127	3,700964
15,59611	0,5697725	770224,8	0,2590945	0,8215805	2015,202
5,299703	0,4914342	70,03317	0,6696293	67,9362	104,4354
62,90217	0,4684657	1017491	0,1691195	0,7753257	86,53317
14,22718	0,3967761	49026,36	0,2429532	0,7154102	2,787377
10,20227	0,488784	300368,2	0,1813665	2,79249	4,207775
13,63434	0,5550024	8423,193	0,4555473	1,736402	2291,164
14,45425	0,7840438	105111,3	0,2638896		
4,911689	0,6762451	9,557315	0,6820221		
58,29683	0,644639	138855,4	0,1722494		
13,18554	0,5459896	6690,549	0,2474495		
9,651196	0,3601373	1700164	0,1913282		
12,89788	0,4089271	47677,5	0,4805686		
13,67351	0,5776854	594957,9	0,2783839		
4,646385	0,498259	54,09693	0,7194828		
55,14794	0,4749715	785958,1	0,1817104		
12,47333	0,4022864	37870,28	0,2610409		
8,814166	0,3808976	1371792	0,1942962		
11,77927	0,4324999	38469,01	0,4880235		
12,48763	0,6109863	480047,1	0,2827024		
4,243412	0,5269814	43,64858	0,7306438		
50,36506	0,5023515	634157,2	0,1845292		
11,39154	0,4254764	30555,97	0,2650903		
7,874576	0,3812537	1023113	0,3067753		
10,5236	0,4329044	28691,04	0,770543		
11,15645	0,6115577	358029,8	0,4463604		
3,791065	0,5274742	32,55408	1,153618		
44,99615	0,5028213	472968,5	0,2913541		
10,1772	0,4258744	22789,32	0,4185526		
10,19353	0,3929995	2301975	0,1967696		
13,62267	0,4462414	64554,04	0,4942361		
14,44187	0,6303988	805556,9	0,2863012		
4,907484	0,5437248	73,24578	0,739945		
58,24692	0,5183123	1064166	0,1868782		
13,17426	0,4389948	51275,33	0,268465		
8,871191	0,387357	1093369	0,2345173		
11,85548	0,4398345	30661,22	0,589049		
12,56842	0,6213478	382615,3	0,3412245		
4,270866	0,5359183	34,78954	0,8818941		
50,69091	0,5108707	505446,8	0,2227284		
11,46524	0,4326919	24354,24	0,3199665		
10,25565	0,5655053	1483260	0,3102902		
13,70568	0,6421175	41594,89	0,7793715		
14,52988	0,9071101	519054,3	0,4514745		
4,937388	0,782391	47,19534	1,166835		
58,60186	0,7458238	685686,9	0,2946923		
13,25453	0,6316901	33038,86	0,4233481		
