# Supplementary material for: Gene expression profiling of patient‐derived pancreatic cancer xenografts predicts sensitivity to the BET bromodomain inhibitor JQ1: implications for individualized medicine efforts
Source: EMBO Mol Med. 2017 Mar 8;9(4):482–97. doi: 10.15252/emmm.201606975 (PMC5376755; doi:10.15252/emmm.201606975)

Figure 4A,B,C chemograms

	JQ1 Concentration (mM	(CRCM17) Mean	SD	N	(CRCM16) Mean	SD	N	(CRCM04) Mean	SD	N	(CRCM110) Mean	SD	N
0,000	0	100	10,92	3	100	7,64	3	100	7,89	3	100	1,35	3
0,001	0,001	90	4,48	3	101	7,02	3	100	4,18	3	89	4,9	3
0,003	0,003	86	3,3	3	102	3,05	3	100	6,18	3	84	2,3	3
0,010	0,01	82	3,85	3	102	2,26	3	98	7,49	3	84	4,77	3
0,030	0,03	82	2,16	3	101	3,64	3	96	9,41	3	86	0,41	3
0,100	0,1	79	2,57	3	98	7,94	3	91	3,83	3	77	1,4	3
0,300	0,3	74	2,55	3	76	0,58	3	78	4,38	3	71	1,25	3
1,000	1	60	0,53	3	51	3,67	3	55	0,43	3	52	1,02	3
3,000	3	51	0,38	3	29	1,08	3	38	0,79	3	42	1,14	3
10,000	10	44	1,6	3	21	0,21	3	24	0,34	3	32	0,69	3
30,000	30	37	0,86	3	14	0,09	3	12	0,13	3	23	0,47	3

	JQ1 Concentration (mM	(CRCM05) Mean	SD	N	(CRCM10) Mean	SD	N	(CRCM11) Mean	SD	N	(CRCM109) Mean	SD	N
0,000	0	100	5,71	3	100	9,76	3	100	9,32	3	100	9,08	3
0,001	0,001	98	5,86	3	99	1,02	3	101	7,02	3	97	6,86	3
0,003	0,003	91	1,89	3	101	4,71	3	95	2,9	3	105	3,76	3
0,010	0,01	93	1,06	3	99	1,52	3	95	5,06	3	100	1,46	3
0,030	0,03	93	3,09	3	101	1,71	3	94	3,74	3	104	2,41	3
0,100	0,1	89	0,96	3	100	4,68	3	91	3,57	3	97	3,51	3
0,300	0,3	83	3,13	3	92	3,53	3	83	0,89	3	89	1,38	3
1,000	1	70	1,09	3	86	0,59	3	69	2,1	3	83	5,76	3
3,000	3	61	1,41	3	75	0,42	3	59	2,1	3	68	1,32	3
10,000	10	55	0,11	3	70	2,28	3	59	1,39	3	64	3,03	3
30,000	30	50	0,91	3	65	1,38	3	55	0,67	3	48	0,65	3

figure 4D spheroids assay

MYC low		MYC high	
DMSO	JQ1 (2µM)	DMSO	JQ1 (2µM)
86,83876	98,67584	88,33624	58,7326
100,2606	66,09727	83,43211	51,31942
84,67612	75,6963	102,945	49,91767
88,29445	75,83363	106,2216	53,19592
86,40707	77,73877	126,8207	56,25889
124,1886	135,6698	105,9222	56,52087
109,7308	86,33214	119,2664	47,15796
88,92326	69,94681	103,4209	64,49735
86,83876	256	88,33624	33,74336
81,12198	67,34264	129,6242	32,83791
75,30049	56,34584	132,5938	33,80598
86,00406	59,71405	106,4214	40,19901
91,30586	52,46368	121,864	45,27991
110,6883	45,46173	113,1813	43,87851
107,812	71,49242	95,83772	42,53554
90,41254	68,27697	91,92612	41,73729
86,83876	69,70473	88,33624	39,95705
95,02122	60,61635	92,11407	37,36454
114,7981	81,55502	68,18607	45,92672
124,5452	81,39976	70,98103	50,39285
139,7758	120,611	84,74858	49,97701
126,3671	86,22461	83,49	36,30051
137,7167	76,62412	101,0047	46,12014
76,13335	89,24203	94,98939	33,89827

figure 4E densitometry Western blot

				**normalisation DMSO = 1**		
	**MYC/Actin**					
**DMSO**	0,73648784	0,73028444	0,78308616	1	1	1
**100 nM**	0,49307371	0,47795068	0,52436556	0,66949334	0,65447194	0,66961414
**250 nM**	0,50308586	0,49665425	0,54147803	0,6830878	0,68008329	0,69146673
**500 nM**	0,33728148	0,31892923	0,36787666	0,45795933	0,43671918	0,46977802
**1000 nM**	0,29555753	0,28686861	0,33496337	0,40130674	0,39281764	0,42774778
**2000 nM**	0,21431798	0,20733268	0,24543298	0,29100002	0,28390675	0,3134176
	**p27/Actin**					
**DMSO**	0,34180971	0,33964859	0,3485488	1	1	1
**100 nM**	0,3779941	0,37749772	0,38076626	1,10586123	1,11143615	1,09243311
**250 nM**	0,59921737	0,59375872	0,59629197	1,75307301	1,74815597	1,71078473
**500 nM**	0,5622749	0,55724952	0,55610137	1,64499396	1,64066489	1,59547635
**1000 nM**	0,69527442	0,69119709	0,69431461	2,03409796	2,03503591	1,99201548
**2000 nM**	0,6573687	0,65093491	0,67611388	1,92320082	1,91649524	1,93979686
	**CAS3/Actin**					
**DMSO**	0,04957735	0,04590744	0,06102248	1	1	1
**100 nM**	0,11034718	0,10614516	0,12488895	2,22575792	2,31215588	2,04660559
**250 nM**	0,12338484	0,1180598	0,14301975	2,48873404	2,57169199	2,3437222
**500 nM**	0,3824577	0,37766744	0,41355253	7,71436315	8,22671536	6,77705183
**1000 nM**	1,09666435	1,0935521	1,14402136	22,1202687	23,8208032	18,7475387
**2000 nM**	1,34567988	1,32856527	1,41241617	27,1430368	28,9400861	23,1458325

Figure 4. E : source data for western blots

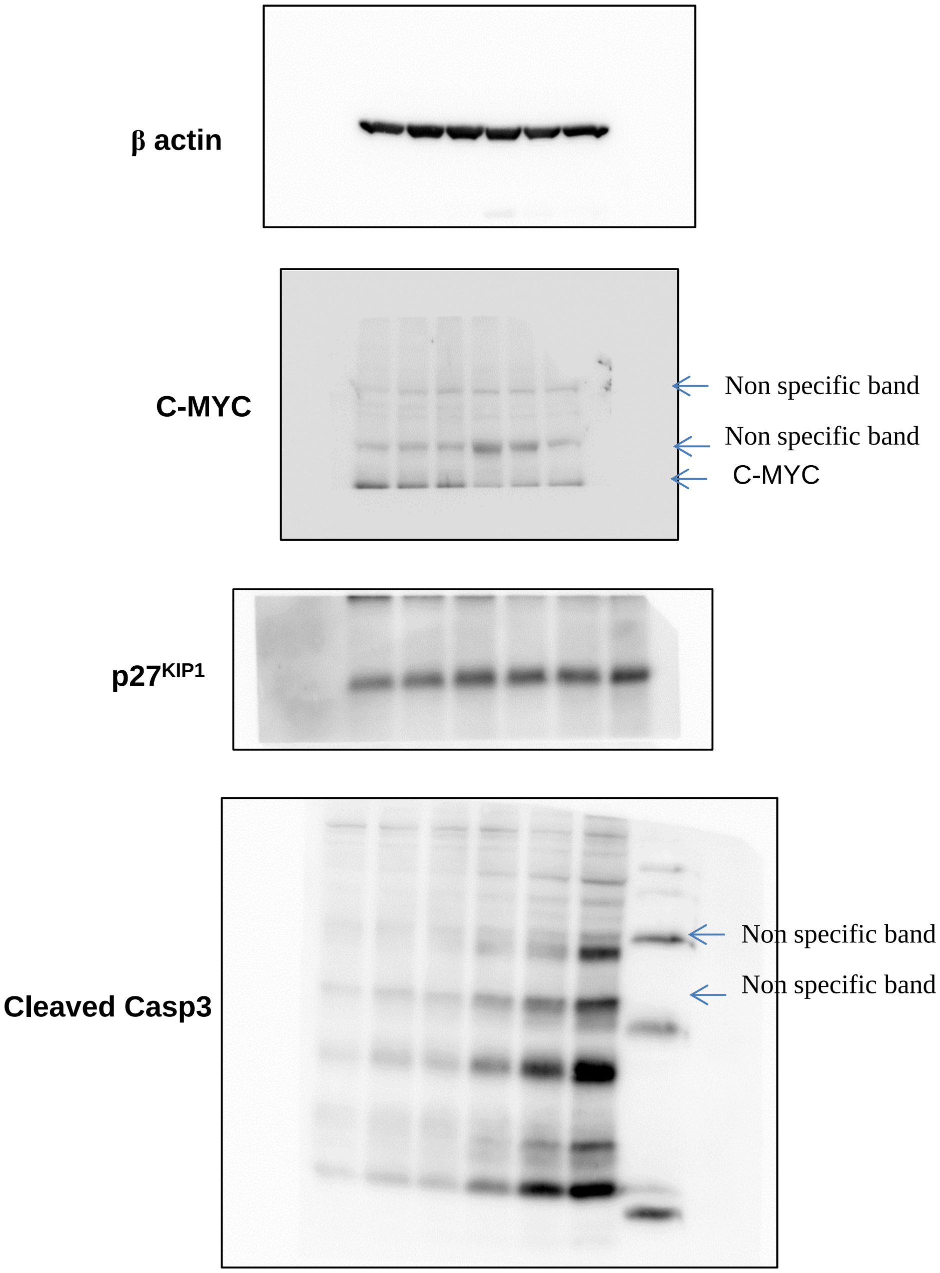

Supplement: Supplementary file 13 — Source Data for Figure 4 [file EMMM-9-482-s012.pdf]
