# Supplementary material for: Gene expression profiling of patient‐derived pancreatic cancer xenografts predicts sensitivity to the BET bromodomain inhibitor JQ1: implications for individualized medicine efforts
Source: EMBO Mol Med. 2017 Mar 8;9(4):482–97. doi: 10.15252/emmm.201606975 (PMC5376755; doi:10.15252/emmm.201606975)
Supplement: Supplementary file 14 — Source Data for Figure 5 [file EMMM-9-482-s013.pdf]

figure 5A

	CRCM23	CRCM43	CRCM26	CRCM50	CRCM19	CRCM21	CRCM108	CRCM25	CRCM28	CRCM112	CRCM30	CRCM114	CRCM29	CRCM116	CRCM34	CRCM42
**ratio 1**	0,156156	2,370498	1,049276	1,466825	7,19294	0,4181563	0,1945823	2,93909	0,5167307	0,302968	689,4346	2,549435	0,2312796	21,66877	0,4920731	0,7340868
**ratio 2**	0,1282614	0,7876627	1,937338	4,020132	2,217354	0,8076025	0,5522882	1,456906	0,2544413	0,1326533	13974,42	1,954206	0,2369239	15,79959	1,197198	2,471892
**ratio 3**	0,151354	1,032379	0,7841663	3,055264	3,479421	0,6817214	0,407329	0,9771314	0,6989793	0,875736	13,97555	3,905201	0,2023633	2,907546	1,603693	0,3189597
**ratio 4**	0,2578239	1,056035	1,982823	5,581858	2,180429	0,9319479	0,199792	0,4627917	0,8418571	0,7407762	5,69702	8,330665	0,1255493	2,691082	1,209181	0,3752898
**ratio 5**	0,1440691	0,6265905	1,451414	2,197978	6,065021	0,6686717	0,4459885	1,6121	0,3496595	0,3635949	4868445	6,099977	0,06510754	4296388	1,023558	1,679146
**ratio 6**	0,1726163	2,124032	4,920038	0,7659152	0,7853044	0,07564653	0,05147373	0,3546287	0,08277048	0,3264786	430,7849	1,010666	0,580195	33,13562	0,7086518	1,036251
**ratio 7**	0,1417814	0,7057678	9,084148	2,099147	0,2420844	0,1460993	0,1460993	0,1757893	0,04075667	0,1429473	8731,747	0,7747002	0,5943546	24,16054	1,724127	3,489369
**ratio 8**	0,1673082	0,9250404	3,676945	1,595333	0,3798732	0,1233268	0,1077526	0,1179001	0,1119633	0,9436938	8,732454	1,548128	0,5076548	4,446185	2,309536	0,4502495
**ratio 9**	0,2850012	0,9462371	9,297429	2,914616	0,238053	0,168594	0,05285187	0,05584015	0,1348496	0,798261	3,559715	3,302502	0,3149568	4,115171	1,741384	0,5297661
**ratio 10**	0,1592554	0,5614426	6,805657	1,147693	0,6621615	0,120966	0,1179793	0,194515	0,05600883	0,3918102	3041989	2,418197	0,1633308	6569986	1,474062	2,370313
**ratio 11**	0,06631666	2,777874	0,4875736	1,129399	20,52587	0,3334327	0,381626	0,8366796	0,5905816	0,4137939	119,4159	1,518332	0,2612213	25,60052	0,9537179	1,746498
**ratio 12**	0,05447032	0,9230247	0,9002351	3,095349	6,327472	0,6439725	1,083179	0,4147417	0,2908059	0,181178	2420,488	1,163839	0,2675964	18,66639	2,320365	5,880986
**ratio 13**	0,06427734	1,209796	0,3643836	2,352437	9,928922	0,5435964	0,7988771	0,278163	0,7988771	1,196081	2,420684	2,325768	0,2285615	3,435114	3,10822	0,758851
**ratio 14**	0,1094933	1,237518	0,9213712	4,297819	6,222101	0,743124	0,3918436	0,1317443	0,9621749	1,011752	0,9867722	4,96138	0,1418031	3,179373	2,34359	0,8928685
**ratio 15**	0,0611836	0,7342718	0,6744376	1,69236	17,30722	0,5331907	0,8746984	0,4589215	0,3996327	0,4965982	843256	3,63288	0,07353645	5075958	1,983823	3,994929
**ratio 16**	0,8256037	1,613425	0,4811191	0,4463551	5,044807	0,4161787	0,5674285	0,9078823	2,638014	0,2138806	145,8623	2,154724	2,631786	3,430351	0,5530332	0,6489905
**ratio 17**	0,6781236	0,5361047	0,8883178	1,223327	1,555154	0,8037832	1,610548	0,4500369	1,298973	0,09364676	2956,539	1,65165	2,696014	2,50121	1,345512	2,185347
**ratio 18**	0,8002152	0,7026653	0,3595599	0,9297175	2,440311	0,6784974	1,187827	0,3018351	3,568429	0,6182269	2,956778	3,300587	2,302741	0,4602894	1,802366	0,2819855
**ratio 19**	1,363127	0,7187665	0,909174	1,698561	1,529256	0,9275405	0,5826208	0,1429559	4,297849	0,5229518	1,205307	7,040886	1,428656	0,4260213	1,358979	0,3317857
**ratio 20**	0,7617	0,4264746	0,6655094	0,6688453	4,253736	0,6655094	1,300563	0,4979765	1,785082	0,2566803	1030007	5,155561	0,7408745	680154,9	1,150361	1,484497
**ratio 21**	0,1948766	3,933821	1,895744	1,329245	3,57731	0,2380228	1,093749	0,3808351	1,088294	0,3569458	236,2354	2,270111	0,2951796	31,65287	0,9510666	1,296708
**ratio 22**	0,1600652	1,30712	3,500221	3,643067	1,102771	0,4597033	3,104417	0,1887798	0,5358823	0,1562873	4788,347	1,740096	0,3023834	23,0794	2,313914	4,366407
**ratio 23**	0,1888839	1,713224	1,416766	2,768698	1,730442	0,3880493	2,2896	0,1266126	1,47213	1,03176	4,788735	3,477335	0,2582741	4,247227	3,099579	0,5634179
**ratio 24**	0,3217543	1,752482	3,5824	5,058312	1,084406	0,5304831	1,123033	0,05996662	1,773047	0,8727555	1,952089	7,417931	0,1602372	3,931025	2,337075	0,6629207
**ratio 25**	0,1797927	1,039822	2,622293	1,991821	3,016355	0,3806211	2,506906	0,2088893	0,7364228	0,4283743	1668177	5,431644	0,08309606	6275991	1,978308	2,966082
**ratio 26**	0,1808434	4,548858	1,094039	0,5885321	7,963561	0,6094955	0,3641739	0,5065543	1,464543	0,2376069	268,5031	4,427621	0,3616286	29,90012	1,346977	0,5503848
**ratio 27**	0,1485388	1,511483	2,019987	1,612992	2,454912	1,177144	1,033645	0,2510988	0,7211494	0,1040352	5442,394	3,393882	0,3704541	21,8014	3,27715	1,853312
**ratio 28**	0,1752822	1,98108	0,8176198	1,225859	3,852191	0,9936624	0,7623436	0,1684093	1,98108	0,6868083	5,442834	6,782189	0,3164152	4,01204	4,389872	0,2391414
**ratio 29**	0,2985846	2,026475	2,067413	2,239602	2,414031	1,358387	0,3739243	0,07976248	2,386031	0,5809641	2,218727	14,46792	0,1963088	3,713347	3,309952	0,2813752
**ratio 30**	0,1668457	1,202394	1,513332	0,8818919	6,714801	0,9746413	0,8346976	0,2778467	0,9910214	0,2851544	1896035	10,59387	0,1018021	5928464	2,801838	1,258947
**ratio 31**	0,143555	8,448281	1,149082	0,4511132	4,544706	0,1063482	0,5163157	0,3656653	2,372545	1,105548	145,524	0,3735667	0,3215318	37,53753	1,100034	0,9006317
**ratio 32**	0,1179114	2,807173	2,121616	1,236368	1,400988	0,2053946	1,465473	0,1812601	1,168255	0,4840597	2949,683	0,2863481	0,3293787	27,37015	2,676347	3,032699
**ratio 33**	0,1391405	3,679324	0,8587555	0,9396282	2,198398	0,1733797	1,08083	0,1215693	3,209331	3,195612	2,949922	0,5722259	0,2813316	5,036839	3,585072	0,3913232
**ratio 34**	0,2370189	3,763633	2,171427	1,716667	1,377658	0,2370189	0,5301394	0,05757797	3,865347	2,703136	1,202512	1,220685	0,1745424	4,66185	2,703136	0,4604332
**ratio 35**	0,1324435	2,233123	1,589471	0,6759751	3,832054	0,1700609	1,183411	0,2005686	1,605445	1,326779	1027618	0,8938246	0,09051446	7442775	2,288174	2,0601
**ratio 36**	0,2942386	4,334628	3,496057	0,5361377	5,163609	0,3540333	0,2073464	0,2798884	0,2803553	0,2637424	206,8963	2,186315	1,719816	37,98599	1,096603	0,5869694
**ratio 37**	0,2416779	1,440299	6,454971	1,469395	1,591776	0,6837592	0,588517	0,1387406	0,1380486	0,1154785	4193,661	1,675865	1,761788	27,69714	2,668	1,976503
**ratio 38**	0,2851904	1,88778	2,612746	1,116727	2,497778	0,5771816	0,4340488	0,09305186	0,3792354	0,7623534	4,194	3,348977	1,504792	5,097014	3,573891	0,2550374
**ratio 39**	0,4858077	1,931038	6,606523	2,04022	1,565269	0,7890366	0,2128979	0,04407148	0,4567545	0,644867	1,70965	7,144114	0,9335963	4,717546	2,694705	0,3000785
**ratio 40**	0,2714639	1,145766	4,835931	0,803381	4,353908	0,566133	0,4752443	0,1535197	0,1897098	0,3165199	1460999	5,231148	0,4841457	7531695	2,281038	1,34263
**ratio 41**	0,5240987	5,578527	2,07285	0,3863254	4,301332	0,4985381	0,4421636	0,5188838	0,8083647	0,2406271	397,6185	2,639246	0,7265072	35,18054	0,2924367	0,8829321
**ratio 42**	0,4304774	1,853618	3,827223	1,058804	1,325964	0,9628473	1,255005	0,2572105	0,3980436	0,1053576	8059,484	2,023047	0,7442374	25,65157	0,7114891	2,973099
**ratio 43**	0,507982	2,429513	1,549126	0,804681	2,080672	0,8127682	0,9256035	0,1725084	1,093471	0,6955381	8,060137	4,042772	0,635674	4,720575	0,9530676	0,3836328
**ratio 44**	0,8653221	2,485183	3,91708	1,470124	1,303883	1,111096	0,4540021	0,0817039	1,316987	0,5883486	3,28565	8,624132	0,3943819	4,369132	0,7186106	0,4513846
**ratio 45**	0,4835322	1,474565	2,867277	0,5788932	3,626843	0,7972099	1,013452	0,2846095	0,5470014	0,288779	2807784	6,314864	0,2045191	6975442	0,608296	2,019614
**ratio 46**	0,3609799	2,194747	3,340321	0,5718189	1,692263	0,5604961	0,2278807	1,123071	0,2975222	0,3591204	330,5977	1,538645	0,6913579	18,65105	0,7053243	1,38528
**ratio 47**	0,296497	0,7292649	6,167428	1,567186	0,521671	1,082509	0,6468	0,5567059	0,1465017	0,1572394	6701,014	1,17941	0,7082304	13,59924	1,716031	4,664657
**ratio 48**	0,3498793	0,9558378	2,496358	1,191047	0,818594	0,9137785	0,4770342	0,3733768	0,402457	1,038046	6,701557	2,356883	0,6049194	2,502624	2,298691	0,6019023
**ratio 49**	0,596002	0,9777402	6,312228	2,176001	0,5129837	1,249182	0,233982	0,1768398	0,4847229	0,8780723	2,731835	5,027756	0,3753012	2,316306	1,733207	0,7082017
**ratio 50**	0,3330392	0,5801347	4,62051	0,8568478	1,426901	0,8962866	0,5223095	0,6160082	0,2013263	0,430984	2334517	3,681483	0,1946243	3698048	1,467141	3,168681

figure 5B,C, D

	01120F (CRCM114)			01.072 (CRCM116)			02.097 (CRCM21)			02.116 (CRCM112)			AU-IPC (CRCM34)			02.071(CRCM28		
concentration	Mean	SD	N	Mean	SD	N	Mean	SD	N	Mean	SD	N	Mean	SD	N	Mean	SD	N
0	100	1,95	3	100	6,6	3	100	9,56	3	100	2,58	3	100	7,62	3	100	4,03	3
0,001	90	7,51	3	99	2,6	3	100	1,52	3	96	5,12	3	100	6,63	3	105	3,34	3
0,003	88	4,85	3	101	3,91	3	100	1,23	3	95	3,61	3	97	4,28	3	103	4,17	3
0,01	84	2,42	3	97	2,5	3	95	7,61	3	94	3,81	3	98	1,89	3	100	5,46	3
0,03	89	6,21	3	98	1,96	3	97	4,79	3	93	2,06	3	96	6,59	3	101	8,22	3
0,1	88	4,21	3	96	4,23	3	93	1,95	3	93	3,4	3	88	5,1	3	101	5,32	3
0,3	75	3,08	3	87	2,23	3	89	2,21	3	91	3,07	3	82	2,02	3	96	8,45	3
1	63	1,95	3	71	0,79	3	78	1,55	3	88	1,04	3	68	1,96	3	92	4,9	3
3	47	0,77	3	54	1,36	3	67	0,69	3	87	1,79	3	58	2,52	3	88	1,08	3
10	36	0,14	3	40	0,45	3	61	1,72	3	81	1,54	3	50	0,66	3	84	4,19	3
30	30	0,41	3	27	1,15	3	51	0,97	3	59	1,02	3	40	0,89	3	79	0,5	3
