# Supplementary material for: Gene expression profiling of patient‐derived pancreatic cancer xenografts predicts sensitivity to the BET bromodomain inhibitor JQ1: implications for individualized medicine efforts
Source: EMBO Mol Med. 2017 Mar 8;9(4):482–97. doi: 10.15252/emmm.201606975 (PMC5376755; doi:10.15252/emmm.201606975)
Supplement: Supplementary file 15 — Source Data for Figure 6 [file EMMM-9-482-s014.pdf]

CRCM16 n=4

days of treatment	Vehicle				JQ1			
1	100	95	108	88	100	98	95	101
3	103	100	112	105	100	100	99	102
7	156	166	132	152	102	109	105	110
10	173	170	136	184	122	128	140	115
14	250	212	232	300	169	120	140	166
17	252	350	230	390	120	143	250	159
21	550	365	678	598	150	198	350	150
24	380	666	856	962	350	257	552	220
28	886	1450	963	756	520	268	632	220

CRCM05 n=4

Days of treatment	Vehicle				JQ1			
1	100	93	111	82	100	98	95	101
3	103	100	112	85	100	100	96	102
7	122	102	166	96	102	109	105	110
10	153	132	170	132	122	128	140	115
14	250	212	232	300	169	155	185	185
17	335	350	423	390	366	166	200	387
21	550	365	678	598	450	350	345	478
24	598	666	856	620	500	450	250	650
28	621	1230	850	620	502	452	256	856

CRCM04

Days of treatment	Vehicle			JQ1		
1	100	95	108	100	98	95
3	125	111	112	100	100	99
7	185	166	190	102	109	105
10	218	250	275	135	128	120
14	289	334	402	169	120	140
17	409	530	623	180	143	203
21	432	648	753	190	198	289
24	745	1200	923	196	257	423
28	769	1450	1400	396	300	589

CRCM10 n=3

Days of treatment	Vehicle			JQ1		
1	100	93	111	100	98	95
3	103	95	112	100	100	96
7	122	99	166	102	109	105
10	153	108	170	105	112	100
14	256	350	301	127	118	108
17	302	358	520	102	100	356
21	560	845	498	139	365	456
24	568	968	530	201	369	568
28	1230	732	756	256	458	780

CRCM114 n=3

Days of treatment	Vehicle			JQ1		
1	98	95	102	100	98	112
3	125	111	112	127	100	153
7	185	166	250	175	189	208
10	350	450	275	179	258	265
14	350	532	402	169	253	256
17	409	530	623	123	250	289
21	432	536	753	128	263	302
24	745	1200	923	196	257	423
28	1002	1450	1400	396	300	589

CRCM116 n=3

Days of treatment	Vehicle			JQ1		
1	98	95	102	100	98	112
3	225	189	320	230	117	189
7	356	562	760	456	237	389
10	587	780	964	567	230	456
14	882	1035	1320	786	368	569
17	1006	1234	1620	789	397	652
21	1298	1289	1875	821	432	687
24	1560	1297	1974	856	487	812
28	1890	1320	2130	1200	498	847

CRCM109 n=3

Days of treatment	Vehicle			JQ1		
1	100	89	123	100	98	78
3	256	112	189	124	145	89
7	456	156	194	320	245	245
10	569	456	394	357	489	487
14	659	560	402	554	420	489
17	889	623	587	689	489	356
21	1023	780	735	589	802	587
24	1020	845	740	610	806	502
28	1130	980	750	498	860	687

CRCM112 n=3

Days of treatment	Vehicle			JQ1		
1	100	89	123	100	98	78
3	256	112	189	124	145	89
7	321	345	208	156	158	145
10	432	356	212	189	230	134
14	793	708	540	265	560	135
17	862	776	617	320	682	278
21	1095	877	686	380	794	468
24	1106	982	719	384	624	754
28	1186	994	686	420	668	835
